# Cystic Chromoblastomycosis: An Unusual Presentation and Diagnostic Challenge

**DOI:** 10.7759/cureus.101558

**Published:** 2026-01-14

**Authors:** Diti P Patel, Nishad S Kosaraju, Bishr A Al Dabagh

**Affiliations:** 1 Medicine, Edward Via College of Osteopathic Medicine (VCOM) Carolinas Campus, Spartanburg, USA; 2 Dermatology, Fora Dermatology, Mooresville, USA

**Keywords:** atypical presentation, chromoblastomycosis, cutaneous fungal infection, cystic, muriform bodies

## Abstract

Chromoblastomycosis (CBM), also known as chromomycosis, is a rare, granulomatous fungal infection caused primarily by pigmented fungi. It typically affects the skin and subcutaneous tissue and is usually acquired through traumatic implantation of fungal spores from contaminated soil, wood, or vegetation. The most common manifestations are verrucous or nodular lesions.

Cystic forms are rare and can mimic other dermatologic conditions, leading to diagnostic challenges. We report a case of CBM in a 43-year-old male presenting with an unusual cystic lesion.

## Introduction

Diagnosing chromoblastomycosis (CBM) can be challenging, particularly when it presents in a manner that deviates from the typical clinical pattern, as seen with the cystic lesion described in this case. CBM is most commonly caused by the fungal species *Fonsecaea pedrosoi*, *Cladophialophora carrionii*, and *Phialophora verrucosa* [1-3]. The WHO classifies CBM as a neglected tropical and occupational disease, as agricultural laborers in low-income tropical regions are most frequently affected [2]. The infection typically occurs on the limbs, particularly the lower limbs and feet [1]. CBM usually begins as a pink papule that gradually progresses into plaques, nodules, or verrucous lesions, sometimes developing into cauliflower-like masses [1]. However, variation in its presentation can mimic other conditions such as tinea, psoriasis, verrucae, or, as in this case, an epidermal inclusion cyst [4]. Although the infection is typically localized, it can spread through scratching. CBM lesions are typically asymptomatic, though they can be mildly pruritic. Symptoms usually arise only if complications occur, making early diagnosis crucial. CBM generally progresses slowly over months to years, and if left untreated, can lead to complications such as lymphedema, secondary bacterial infections, squamous cell carcinoma, tissue fibrosis, and mobility issues [1,4]. However, achieving a high index of suspicion for an accurate diagnosis can be challenging due to variability in clinical presentation. The diagnosis is confirmed by the identification of muriform bodies within multinucleated giant cells on pathology [1].

## Case presentation

A 43-year-old male presented to the dermatology clinic with a lesion on his right index proximal interphalangeal joint. The lesion had been present for 30 years. He first noticed it following a splinter injury to the finger. The patient endorsed itching and pain associated with the bump, which had been gradually increasing in size in recent years, prompting a visit to the clinic. He had not attempted any treatments for the lesion and denied any other pertinent past medical history, being otherwise healthy.

**Figure 1:**
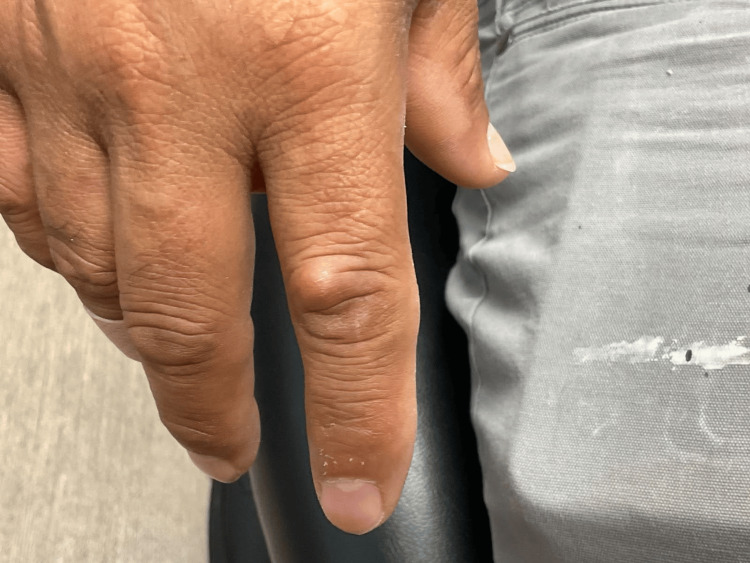
Firm, non-erythematous, and non-tender lesion over the proximal interphalangeal joint of the right index finger, measuring approximately 0.7 × 0.7 cm.

On physical examination, a skin-colored, cystic papule was noted over the proximal interphalangeal joint of the right index finger, measuring approximately 0.7 × 0.7 cm. The lesion was firm, non-erythematous, and non-tender (Figure 1).

**Figure 2:**
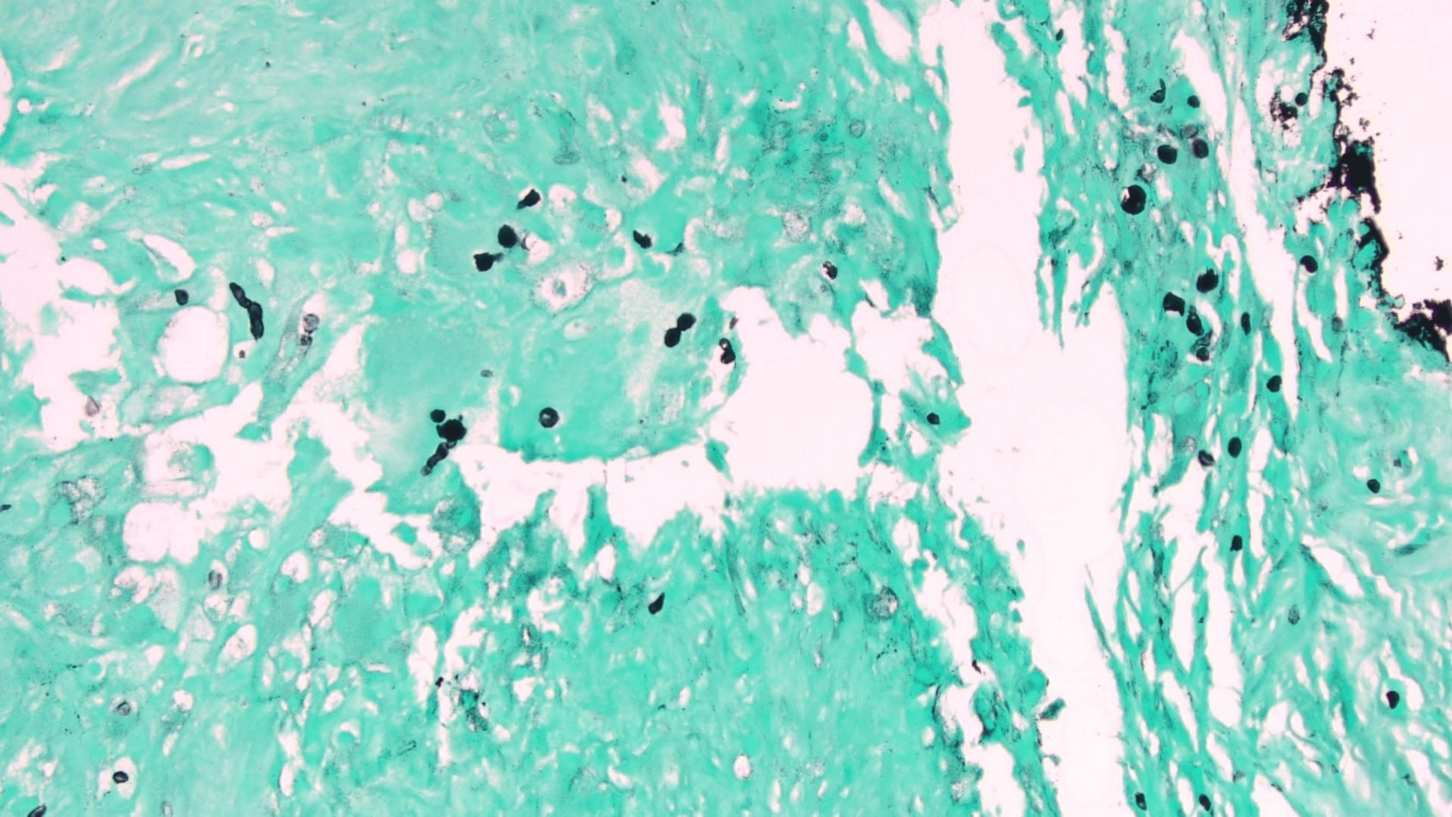
Histopathology of the biopsy site at 200× magnification (H&E and Grocott methenamine silver stains) shows mixed granulomatous inflammation with palisading histiocytes around necrotic eosinophilic material and thick-walled, dark fungal cells (muriform bodies).

The differential diagnosis included an epidermal inclusion cyst and pilomatrixoma, given the lesion’s long-standing duration, firm consistency, slow growth, and cystic appearance on physical examination. A surgical excision was performed, revealing areas of mixed granulomatous inflammation, with palisading histiocytes around necrotic eosinophilic material, consistent with CBM. Granulomatous inflammation with palisading histiocytes may also be seen in entities such as foreign body granulomas, rheumatoid nodules, deep fungal infections, and mycobacterial infections; however, the identification of muriform bodies is pathognomonic for CBM. Muriform bodies, some in short chains with slight brown pigmentation, were observed and highlighted with Periodic acid-Schiff-Harris hematoxylin and Grocott methenamine silver stains (Figure 2).

Fungal culture was not performed. The lesion was successfully excised, and the patient was instructed to follow up for further evaluation. At his 4-month follow-up, there was no recurrence of the lesion, and the patient reported feeling well with no further symptoms. No additional treatment was required. The patient was instructed to monitor his finger and return to the clinic if there was any recurrence. The diagnosis of CBM was established histologically by the presence of muriform bodies within granulomatous inflammation.

In this case, the patient’s solitary, localized lesion was successfully cleared by excision with confirmation of clear margins. However, had the infection spread to form satellite lesions, treatment would likely have included chemotherapy, immunotherapy, cryotherapy, or systemic antifungal therapy with itraconazole or terbinafine [1].

## Discussion

This case highlights the diagnostic challenge of CBM when it presents outside its classic clinical phenotype and underscores the importance of histopathologic evaluation in long-standing, indolent cutaneous lesions. Although CBM typically manifests as slowly progressive, verrucous or cauliflower-like plaques with hyperpigmentation on the lower extremities, this patient instead developed a solitary, non-pigmented cystic nodule that remained localized for more than three decades [1,4]. Such an atypical presentation likely contributed to the prolonged delay in diagnosis. CBM is a rare deep fungal infection in non-endemic regions and is most often associated with tropical climates, agricultural exposure, and lower-extremity involvement, which may lower clinical suspicion when these features are absent.

Several factors may have contributed to this unusual morphology. The localized, nodular growth pattern suggests a balance between fungal persistence and host immune containment, in which deeper dermal inoculation from trauma may have limited superficial epidermal involvement and prevented the epidermal hyperplasia and papillomatous architecture characteristic of classic CBM [1]. The absence of lymphatic dissemination or satellite lesions further supports a localized host response capable of containing the infection over time.

Histopathologic examination was pivotal in establishing the diagnosis. The identification of pigmented muriform (sclerotic) cells within a granulomatous inflammatory background represents the histopathologic hallmark of CBM and remains the most definitive diagnostic feature, particularly when fungal cultures are unavailable or negative [1]. These thick-walled, septate fungal elements reflect the tissue-adapted form of dematiaceous fungi and differentiate CBM from other deep fungal infections. Recognition of muriform cells is therefore critical, especially in atypical presentations lacking classic clinical features.

The localized nature of this infection allowed for curative surgical excision without systemic antifungal therapy, contrasting with advanced CBM, which often requires prolonged multimodal treatment, including azole antifungals, cryotherapy, or adjunctive immunotherapy [1]. Untreated or progressive disease may lead to complications such as lymphedema, secondary bacterial infections, or malignant transformation to squamous cell carcinoma [1,4].

This case reinforces the need for clinicians to broaden the differential diagnosis of chronic, post-traumatic cutaneous nodules, even when lesions lack classical pigmentation, verrucous morphology, or epidemiologic risk factors. Early biopsy and careful histopathologic review are essential to prevent diagnostic delay, disease progression, and long-term complications.

## Conclusions

In conclusion, this case highlights an atypical presentation of CBM that could have easily been overlooked and left untreated. Unlike the more commonly reported pigmented, verrucous lesions found on the lower extremities, this patient’s biopsy-confirmed CBM presented as a relatively asymptomatic, firm, non-erythematous, non-tender cystic papule on the finger. The skin-colored lesion, which gradually enlarged over 30 years, resembled an epidermal inclusion cyst, potentially leading to misdiagnosis. Given the rarity of cystic forms of CBM, clinicians should remain vigilant when evaluating patients with a history of trauma, particularly those from endemic regions, as early manifestations of the disease can appear as seemingly benign lesions. Prompt recognition of such cases is essential to avoid diagnostic delays and prevent disease progression and associated complications.
